# Factors associated with the non-use of insecticide-treated nets in Rwandan children

**DOI:** 10.1186/s12936-016-1403-6

**Published:** 2016-07-12

**Authors:** Monique Murindahabi Ruyange, Jeanine Condo, Corine Karema, Agnes Binagwaho, Alphonse Rukundo, Yvette Muyirukazi

**Affiliations:** Malaria and Other Parasitic Diseases Division-RBC, Ministry of Health, Kigali, Gasabo Republic of Rwanda; Ministry of Health, Kigali, Gasabo Republic of Rwanda; Department of Global Health and Social Medicine, Harvard Medical School, Boston, MA USA; Dartmouth College, Geisel School of Medicine, Hanover, NH USA; School of Public Health, College of Medicine and Health Science, University of Rwanda, Kigali, Gasabo Republic of Rwanda

**Keywords:** Malaria, Prevention, ITN, Children under 5 years

## Abstract

**Background:**

Insecticide-treated bed nets (ITNs) are highly effective in reducing malaria burden when used properly. However, factors related to individuals, households and community may influence how ITNs are used for malaria control. The study examined influences exerted at these levels to determine if they are associated with ITN non-use among children under 5 years of age in Rwanda.

**Methods:**

Using data from the 2010 Rwanda Demographic Health Survey, the investigation was done on the factors associated with ITN non-use among children under 5 years. Descriptive statistics as well as univariate and multilevel logistic regression analyses were used to identify factors associated with ITN non-use.

**Results:**

Responses from a total of 6173 women aged 15–49 years living in 492 villages were included in the analysis. Risk factors for children not utilizing ITNs (25 %) included: (Odds ratio [95 % confidence interval]) households with more than five members (1.42 [1.23–1.63]), employed mother (1.33 [1.06–1.66]), and lower household altitude (1.36 [1.14–1.61]). Protective risk factors for ITN use included households with more than three nets (0.39 [0.33–0.47]), mothers who attended one to four visits at antenatal clinics during pregnancy (0.45 [0.29–0.69]), more than four antenatal clinic visits during pregnancy (0.39 [0.21–0.70]), mothers married or living with partner (0.43 [0.36–0.52]), mothers with any education level (0.77 [0.65–0.91]), and households with higher community wealth quintile (0.71 [0.59–0.84]).

**Conclusions:**

Rwanda has achieved high coverage of ITN use and proper use has contributed to a decline in malaria in Rwanda; however, maintaining universal ITN coverage is not enough to protect citizens from this disease. Risk factors related to ITN non-use at individual, household and community level include poverty, education, birth spacing, and antenatal clinic attendance. There is a need to address findings with strategies to mitigate the non-use of ITNs for effective malaria prevention in Rwanda.

## Background

Malaria is the leading cause of morbidity and mortality in sub-Saharan Africa for children under 5 years of age (U5) and was responsible for approximately 6.5 % of deaths among U5s in Rwanda in 2011 [1, 2]. Following the scale-up of comprehensive malaria control interventions between 2007 and 2010, the prevalence of malaria parasitaemia measured by household surveys declined from 2.6 to 1.4 % in children between six and 59 months of age, and U5 mortality declined by 42 % from 133 deaths to 76 per 1000 live births [3].

Insecticide-treated bed nets (ITNs) are an important malaria prevention intervention in reducing childhood malaria morbidity and mortality. Repeated studies have been shown that ITNs are the most cost-effective measure to reduce malaria transmission in developing countries [4, 5]. In 2010, Rwanda distributed approximately 4.1 million ITNs during a universal coverage campaign with the goal of one ITN per two people, thereby covering all sleeping areas in each household. This campaign resulted in an increase of 25 % of U5 sleeping under an ITN compared to 2007–2008 [3, 10, 15]. The 2010 universal ITN campaign resulted in 75 % of U5 sleeping under a mosquito net, relatively high compared to other sub-Saharan countries, but nonetheless below Rwanda’s national and international target of 80 % [3].

A number of studies have examined factors associated with ITN non-use among U5 in sub-Saharan Africa [6, 8–13]. However, the focus of these studies have been on individual-level factors, and only a few have examined community-level factors [7]. The 2010 Rwanda Demographic Health Survey (DHS) data were analysed to identify social and ecological factors, including individual, household and community factors associated with non-use of ITNs among U5s in order to inform national malaria control programmes on areas of focus for increase of ITN use among U5s.

## Methods

### Study setting

Rwanda is situated in East Africa, immediately south of the Equator [3]. Uganda is to the north, Tanzania to the east, the Democratic Republic of the Congo to the west, and Burundi to the south of the country. Rwanda’s average elevation varies between 1500 and 2000 m. Rwanda enjoys a temperate, sub-equatorial climate with average yearly temperatures of around 18.5 °C. The average annual rainfall is 1250 mm and occurs in two rainy seasons of differing lengths (February to June and September to December), alternating with one long (June to September) and one short dry season (December to February). Rwanda has a dense network of rivers and streams, and several lakes surrounded by wetlands [3].

### Study design and sample size

The 2010 Rwanda DHS, conducted by the Rwanda National Institute of Statistics in collaboration with the Ministry of Health, collected data on 13,671 women of reproductive age and 6329 men aged 15–59 years. Methods and data collection procedures have been published previously [3]. Sites were selected at random and comprised 492 sampling clusters. In selecting sites, the sampling scheme accounted for rural and urban areas and for different regions in the country. For this study, communities were based on sharing a common primary sample unit (PSU) within the Rwanda DHS data. Data from female respondents were aggregated and used for statistical analysis as described below.

### Measures

Individual child, parent, household, and community characteristics were examined to identify factors that may affect the non-use of ITNs among U5s. The term community was used to describe clustering within the same geographical living environment. These characteristics included: (1) the age and sex of the child; (2) the age, education level, employment status, marital status, and antenatal (ANC) attendance of the mother; (3) income or socio-economic status, rural or urban setting, the number of ITNs owned, the number of U5s in the household, and the total number of the household; and, (4) wealth index, education level and altitude (either lower or higher than 1600 m) of the community.

The education level was categorized into: no formal education, primary education (up to age 6 years), secondary, and post-secondary education. The wealth index was categorized into three groups based on household income: poorest, poor/middle, high, and used a proxy for socio-economic status of a household.

### Statistical analysis

#### Descriptive analyses

Pearson’s Chi squared test was used for analysing contingency tables. Data were weighted, based on probability of selection and non-response. Pooled sample weights were used for descriptive statistics. STATA 11 for Windows (STATA, College Station, TX, USA) was used for the analysis.

#### Multivariate modeling approaches

Multilevel logistic regression models were used to examine factors associated with ITN non-use among U5s. A four-level model was specified and each was tested by fitting univariate logistic regression models and estimating odds ratios (OR). Those variables, having a *P* value of less than 0.05, were retained and a backward regression method was applied in order to refine the model.

Five linear regression models were constructed using characteristics from: (1) child; (2) mother; (3) household; (4) community; and, (5) combination of the four. The measures of association (fixed-effects) were reported as ORs with their 95 % confidence intervals. The measures of variation (random effects) included variance and intra-cluster correlation. The multilevel models were fitted with MLwiN 2.24 software. The statistical significance of covariates was calculated using the Wald test. All significance tests were two-tailed and statistical significance was defined at the 5 % alpha level.

## Results

### Descriptive results

For this analysis, information on 6173 female respondents was pooled into one dataset. Nearly two-thirds of the respondents had children aged more than 23 months (64.7 %), and the majority of respondents lived in rural areas (85.8 %). Many households (72.2 %) owned one or two ITNs and almost all households had one or two U5s (92.6 %). The majority of respondents were married or living with a partner (84.9 %) and were not employed or had an agricultural occupation (86.7 %). Of those who had children, almost all mothers attended one to four ANC visits (95.6 %). At the community level, 56.1 % of households were categorized in middle or higher wealth index, 81.0 % of mothers had at least some primary education and 58.8 % were living in area at an altitude of more than 1600 m (Table 1).

**Table 1:** Social-ecological model grouping of characteristics from 6173 study participants

Level 1: individual	*n* (%)
Children age (months)	
≤12	1110 (17.98)
13–23	1072 (17.37)
>23	3991 (64.65)
Children sex	
Female	2995 (48.52)
Male	3178 (51.48)
Children birth weight (g)	
≥2500	5926 (96)
<2500	247 (4)

Level 2: household	*n* (%)
Residence	
Urban	874 (14.16)
Rural	5299 (85.84)
Number of nets (nets)	
≤2	4498 (72.87)
≥3	1675 (27.13)
Number of household members	
<5	3638 (58.93)
>5	2535 (41.07)
Number of children under 5 years	
≤2	5713 (92.55)
>2	460 (7.45)
Number of living children (children)	
≤2	2726 (44.16)
>2	3447 (55.84)
Wealth index	
Poorest and poor	2712 (43.93)
Middle and higher	3461 (56.07)
Number of rooms used for sleeping	
≤2	4338 (70.62)
≥3	1805 (29.38)

Level 3: mother	*n* (%)
Marital status	
Never in union, widowed, divorced	932 (15.1)
No longer living together	
Married or living with partner	5241 (84.9)
Occupation	
Not employed or agricultural	5345 (86.69)
Employed	821 (13.31)
Education	
No education	1173 (19)
Any education	5000 (81)
ANC visits during pregnancy	
No ANC	103 (1.73)
1 to 4 ANC visits	5685 (95.56)
>4 ANC visits	161 (2.71)

Level 4: community	*n* (%)
Community wealth index	
Poorest and poor	2712 (43.93)
Middle and higher	3461 (56.07)
Community education level	
No education	1173 (19)
Any education	5000 (81)
Altitude of Cluster in meters (m)	
<1600	2546 (41.24)
>1600	3627 (58.76)

### Association between contextual and individual factors and ITN non-use among U5s

A total of 6173 women aged between 15 and 59 years from 12,540 surveyed households were included in the analysis. Of the 15 independent variables analysed, nine were found to be associated with non-use of ITN at p ≤ 0.05 and were included in the final model (Table 2). The statistically significant variables were: (1) mother level—marital status (0.46 [0.39–0.55]), the occupation (1.36 [1.10–1.66]); (2) household level—urban/rural (1.06 [0.86–1.30]), number of available nets (0.43 [0.36–0.51]), number of household members (1.42 [1.20–1.66]); (3) community level—socio-economic status (0.73 [0.64–0.84]), education level (0.7 [0.59–0.81]) and altitude (1.37 [1.19–1.56]).

**Table 2:** Univariate and multivariate analysis of determinants of bed net non-use among children under five years, Rwanda DHS 2010

Variables	n (%)	Univariate analysis	Multivariate analysis
		OR (95 % CI)	OR (95 % CI)
Children age (M)			
≤12	279 (23.98)	Ref	Ref
13–23	247 (23.41)	0.93 (0.75–1.15)	0.92 (0.73–1.15)
>23	1019 (25.79)	1.03 (0.87–1.22)	1.02 (0.86–1.22)
Children sex			
Female	745 (24.81)	Ref	Ref
Male	800 (25.27)	0.93 (0.82–1.06)	0.93 (0.81–1.06)
Number of living children (children)			
≤2	648 (23.71)	Ref	Ref
>2	897 (26.1)	1.12 (0.94–1.33)	1.13 (0.94–1.36)
Children birth weight (g)			
≥2500	1482 (25.03)	Ref	Ref
<2500	63 (25.37)	1.27 (0.93–1.73)	1.23 (0.90–1.67)
Mother marital status			
Never in union, widowed, divorced, no longer living together	364 (39.12)	Ref	Ref
Married or living with partner	1181 (22.57)	0.46* (0.39–0.549)	0.44*** (0.37–0.52)
Mother education			
No education	390 (25.24)	Ref	Ref
Any education	1155 (74.76)	0.65* (0.56–0.76)	0.77*** (0.65–0.90)
Mother occupation			
Not employed or agricultural	1332 (25)	Ref	Ref
Employed	211 (25.33)	1.36* (1.10–1.66)	1.34*** (1.08–1.66)
ANC visits			
No ANC	50 (48.21)	Ref	Ref
1 to 4 ANC visits	1383 (24.34)	0.50 (0.33–0.76)	0.51*** (0.33–0.79)
>4 ANC visits	33 (20.59)	0.46 (0.26–0.81)	0.47** (0.26–0.86)
Residence			
Urban	183 (21.43)	Ref	Ref
Rural	1362 (25.56)	1.06** (0.86–1.30)	0.96 (0.74–1.24)
Number of mosquito bed nets (nets)			
≤2	1287 (28.61)	Ref	Ref
≥3	258 (15.29)	0.43* (0.36–0.51)	0.43*** (0.36–0.51)
Number of household members			
<5	861 (23.71)	Ref	Ref
>5	684 (26.98)	1.42* (1.20–1.66)	1.42*** (1.23–1.63)
Number of children U5			
≤2	1435 (25.15)	Ref	Ref
>2	110 (23.74)	0.96 (0.74–1.23)	0.95 (0.73–1.24)
Number of rooms used for sleeping			
≤2	1106 (25.56)	Ref	Ref
≥3	429 (23.58)	1.02 (0.87–1.19)	1.03 (0.87–1.21)
Household wealth index			
Poorest and poor	901 (35.42)	Ref	Ref
Middle and higher	780 (22.50)	0.81* (0.71–0.93)	0.81** (0.70–0.94)
Community wealth index			
Poorest and poor	835 (30.84)	Ref	Ref
Middle and higher	710 (20.39)	0.73* (0.64–0.84)	0.73*** (0.63–0.85)
Community education level			
No educ	390 (32.89)	Ref	Ref
Any educ	1155 (23.17)	0.70* (0.59–0.81)	0.71*** (0.60–0.85)
Altitude of cluster in meters (m)			
<1600	523 (20.67)	Ref	Ref
>1600	1022 (28.15)	1.37* (1.19–1.56)	1.35*** (1.14–1.60)

*** p < 0.01, ** p < 0.05, * p < 0.1

The following variables were not included in the final model (*p* > *0.05*): (1) number of U5s residing in the household; (2) number of rooms used for sleeping available in the household; (3) age of children; (4) gender of children; (5) birth weight of children.

### Determinants of ITN non-use among U5s

Findings from modeling the dependent variables and social-ecological-level factors are shown in Table 2. There is no statistically significant association between the ITN non-use and age (1.02 [0.86–1.22]), gender (0.93 [0.81–1.06]), or birth weight of the children (1.35 [0.98–1.87]). Additionally, children born to a married mother or mother living with a partner (0.43 [0.36–0.52]), born to a mother having some education (0.77 [0.65–0.91]), born to a mother who had 1–4 (0.51 [0.33–0.79]) or more than four ANC visits (0.47 [0.26–0.86]) were more likely to sleep under a bed net compared to children born to a widowed, divorced or employed (or agriculturally employed) mother. It was observed that children living in a household having three or more ITNs (0.43 [0.36–0.51]) were more likely to use them than children who live in a household with two or fewer ITNs. Children living in a household with five or more members (1.39 [1.17–1.65]) were less likely to sleep under an ITN than their counterparts living in household with five members or fewer. Including the dependent variables and the community-level factors in the model revealed that ITNs were significantly highly used in middle and higher wealth index communities (0.73 [0.63–0.85]) and in communities with education levels above primary schooling (0.71 [0.59–0.84]). Children living at altitude more than 1600 m (1.36 [1.14–1.61]) were less likely to use an ITN than their counterparts living at below 1600 m. The result of the full model, including all covariables, is shown in Table 2. In the final model, controlling for all factors, the following remained significantly associated with the odds of not sleeping under a net: mother-level (occupation), household-level (number of household members) and community-level (altitude where the cluster is located).

## Discussion

The study analysed community-level factors that influence ITN non-use among U5s in addition to individual, maternal and household factors. In particular, the analysis suggests that at community level, ITN non-use among U5s were associated with communities with high illiteracy rate, low wealth index and elevation higher than 1600 m. This suggests that children in the same community may be subject to common influences that determine their ITN usage.

The findings of the study agree with previous studies [8] on non-community factors correlated with ITN use among U5s, married or in-union mother, educated mother, mother with an occupation other than agriculture, mother having attended one to four or more than four ANC visits. Rwanda has achieved high coverage of ITN use among pregnant women since 2005, as a result of ITNs being distributed to pregnant women during their first ANC visit [9]. Additionally, children from households with more than three nets or fewer than five members are more likely to use an ITN. As seen in other studies [8], in Rwanda the association between socio-economic status (SES) and ITN non-use was not as clear as some studies found higher SES related to higher ownership and utilization [10], while others did not find such an association. This may be explained by the Rwanda Government 2020 Vision priority for health equity with its strategies of ITN universal coverage and free distribution of ITN to all households in need. Variables, such as occupation and education levels of the household, appeared to be directly associated with ITN use in some studies [10].

The analysis suggests that mothers without marital status (who were not in union, widowed, divorced, or no longer living with a partner) is associated with non-ITN use among U5s. This finding is similar to results from other studies that found children with married mothers or with mothers who were living with their partners were more likely to use ITNs compared to children whose mothers were never married [11, 12].

The low education level of mothers may affect ITN non-use as described by Baume et al. [13] where the mother’s educational level and knowledge were found to be associated with lower ITN use. Other studies found that education level of the head of household was not independently associated with ITN use by U5s [14]. In the analysis, maternal education level is shown to be associated with higher ITN use among U5s.

The analysis also suggests that maternal employment, ANC attendance (one to four visits) and more than four ANC visits increased the likelihood of ITN use by U5s by 1.38-, 1.95- and 2.21-fold, respectively. The few systematic reviews on ITN use among U5s have not shown similar findings [13, 15]. A possible explanation could be that in Rwanda, pregnant women are given ITNs and sensitized on malaria prevention with an emphasis on ITN use during their first ANC visit [9].

The analysis suggests that a U5 is more likely to sleep under an ITN if there are more nets in the household, as shown in other studies [15, 16]. The number of household members was also an important factor predicting ITN use. Children from households with five or more individuals are less likely to use ITNs, which agrees with findings by Siri [17].

The analysis also suggests that DHS data can be analysed for cluster-level factors. The analysis showed that at the cluster-level, low education level and low SES were associated with ITN non-use. In addition, the study showed that children living in high-altitude clusters (> 1600 m) are 1.36 more likely to not use ITNs, which is similar to the results of a survey conducted in Ethiopia on ownership and use of ITNs for malaria prevention [18].

However, the findings conflict with findings from some studies conducted in six African countries which showed that ITN non-use was associated with age [19], which was not statistically significant in the analysis. Gender differences on ITN non-use among U5s was not statistically significant in a study conducted in Ethiopia, Ghana, Mali, Nigeria, Senegal, and Zambia [19], similar to the results of the analysis. Another variable associated with using ITNs was urban/rural residence, which was not statistically significant in the analysis [5].

### Limitations

As discussed in other analyses, using DHS data and considering PSUs (clusters) at community level, results may be biased towards populations in organized communities. In addition, the use of cross-sectional data limits the inferences to associations between independent and dependent variables, not causality. There are also limitations associated with the community-level characteristics used in this analysis. With the exception of urban/rural residence all community-level variables were constructed by aggregating individual-level and household-level characteristics at community level (the PSUs). Therefore, the community contextual variables are actually composite variables, aggregated upwards. There are two potential problems with this approach. First, it could result in multi-colinearity since variables used to derive the community-level contextual variables are also individual-level variables. Second, this approach makes inferences at a higher level based on data collected at a lower level, which could lead to inaccuracies.

## Conclusion

The results of the analysis suggest that mother, household and community-level factors were associated with the ITN non-use among U5s in Rwanda, and suggest that strategies designed to improve ITN use among U5s should address both individual and community-level factors. Based on this analysis, there is a need to ensure increased ownership and use of ITNs in U5s by tackling poverty reduction in the community with possible income-generating cooperatives, strengthening women and girls’ opportunities for education, encouraging Ministry of Health policy on birth spacing and family planning, and improving early and frequent ANC attendance among pregnant women. Many of these strategies are multifactorial, complex and beyond the scope of the health system (i.e., poverty reduction and education) but certainly impact health as observed. Therefore, the recommendation is that health be a priority for sectors other than the health sector, with most importantly the engagement and involvement of community/local leaders along with their communities to identify challenges of non-ITN use and mobilize interventions to mitigate this problem. A final, feasible, real-time recommendation for a national malaria control programme is to advocate and emphasize the integration and utilization of long-lasting ITNs among U5s. These coordinated efforts will increase ITN use among U5s. This would afford Rwanda the opportunity to achieve the international and national malaria control targets on ITN use and continue to reduce malaria burden.

## Abbreviations

ANC: antenatal care services
CI: confidence interval
DHS: Demographic Health Survey
ITN: insecticide-treated bed net
MoH: Ministry of Health
OR: odds ratio
PSU: primary sample unit
U5: children under 5 years
SES: socio-economic status
